# Directional Crystallization of Conjugated Molecules during Coating Processes

**DOI:** 10.3390/molecules28145371

**Published:** 2023-07-13

**Authors:** Detlef-M. Smilgies, Ruipeng Li

**Affiliations:** 1 Center of Advanced Microelectronics Manufacturing (CAMM) and Materials Science and Engineering Program, Binghamton University, Binghamton, NY 13902, USA; 2 R. F. Smith School of Chemical and Biomolecular Engineering, Cornell University, Ithaca, NY 14853, USA; 3 NSLS-II, Brookhaven National Laboratory, Upton, NY 11973, USA; rli@bnl.gov

**Keywords:** conjugated molecules, crystallization, coating techniques

## Abstract

The coating of organic molecules from the solution phase can result in directional crystal growth under certain conditions, even on a smooth isotropic surface and without the need of any kind of graphoexpitaxial preparation of the substrate. Based on reviewing the results from a variety of coating techniques and coating parameters, we identified that it is crucial for the coating speed to match the growth speed of the fastest growing crystal plane to achieve a high degree of directional crystallization.

## 1. Introduction

Coating from the solution phase, a technology featuring low energy consumption and low production cost, is one of the key technologies for the processing of organic semiconductors. Coating methods have evolved from lab-scale methods, such as spin coating to industry-favored roll-to-roll coating methods such as dip coating, blade coating, or slot-die coating. This evolution requires an accurate understanding of the kinetic processes involved in the non-equilibrium crystallization of conjugated molecules in a more complex parameter space. However, the details of coating processes of functional materials are not easy to access without powerful real-time in situ characterization. Synchrotron-based X-ray scattering methods in combination with suitable sample environments have recently been demonstrated to provide such information [1,2,3]. In addition, in situ high-speed cross-polarized optical microscopy has been a powerful tool to probe the formation of the emerging crystalline domains [2,4].

Here, we will focus on the material class of conjugated molecules, such as TIPS-pn (6,13-bis(triisopropyl silanyl ethynyl) pentacene), diF-TES-ADT (2,8-difluoro 5,11-bis(triethylene silanyl ethynyl) anthradithiophene), or C8-BTBT (2,7-dioctyl benzothienobenzothiophene), all of which are small molecules which have recently been shown to display high charge mobilities while being processed from solution at low temperatures between 50 and 100 °C. The charge carrier mobility in a molecular organic semiconductor is determined by the intrinsic, highly anisotropic mobility inside a single crystalline grain and the grain boundaries that the charge carriers have to pass. Hence, the deposition of single crystalline domains across the gate channel of typically a few micrometers in length is of high interest, in order to enhance performance and reproducibility of organic field-effect transistors. Recently, a number of publications have reported directional growth of long crystalline needles which would eliminate grain boundaries from the charge carrier path [5,6,7,8,9,10,11]. Such studies shall be reviewed in the following sections and a suggestion for the growth mechanism shall be given.

## 2. Coating Processes

Common to all deposition processes of conjugated molecules is that there is often a preferential crystallographic plane that grows parallel to the substrate surface while laterally, the grains have either random orientations (so-called 2D powders) or a certain degree of directionality that depends on the details of the deposition process.

**Figure 1:**
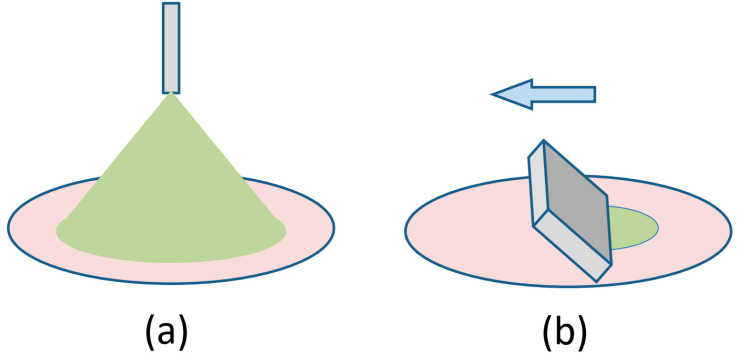
Classification of coating processes. (**a**) Isotropic coating such as drop casting, spray coating (shown), and spin coating result in a random lateral orientation of grains on the substrate. (**b**) Directional coating, such as slot-die coating, blade coating (shown), or dip coating control the shape of the drying line and can result in a preferential lateral orientation of the crystalline domains, as discussed in the text.

Coating processes can be classified as isotropic or directional (see Figure 1). Isotropic growth, i.e., arbitrary lateral grain orientation, has been observed in drop casting, spray coating, and, for the most part, in spin coating. In directional coating processes, mechanical devices or drying geometry impose a straight drying line that sweeps across the substrate as the solvent evaporates. The most sophisticated coating technique of this class is premetered slot-die coating. Here, the amount of material going into the slot-die head and the amount of material deposited on the substrate are precisely controlled; this is an important factor when coating costly designer molecules with typical prices of USD 1000 per gram. A simpler method is self-metered knife coating, also referred to as blade coating, doctor blading, or shear coating, with some minor variation in the details. Finally, there is pen writing and dip coating where the solution is deposited by capillary action. In the following paragraphs, we will focus on such directional coating processes.

Directional coating is a non-equilibrium process involving multiple length scales and time scales. When the solution leaves the coating device, it thins within the meniscus region from the coating gap of typically 100 to 500 µm to about a tenth of this value at the end of the meniscus—this stage can be understood by the hydrodynamic interplay of viscosity and surface tension. The next stage is evaporation which starts as soon as the solution leaves the coating device and continues until the solute solidifies. Typically, conjugated molecules are dissolved at concentrations around 10 mg/mL which corresponds to a volume ratio of solvent to solute of about 100:1. Hence, the film thins by another factor of 100 due to the evaporation of the solvent. The goal for the deposition is to achieve film thicknesses of 50 to 100 nm which constitutes a compromise between reproducibility, reliability, and cost efficiency, with the channel width of an organic field-effect transistor typically being 30 nm.

It is now a matter of the coating regime whether solution deposition, solvent evaporation, and solute crystallization occur simultaneously or can be considered as two independent steps. In the former case, the evaporation regime, the film solidifies at the edge of the meniscus, the drying line. Here the film thickness *h* scales with the coating speed *v* as *v*^−1^ [12]. At faster coating speeds, the final film thickness is determined by the hydrodynamic boundary layer thickness, the well-known Landau–Levich regime, in which *h* scales as *v*^2/3^ [12,13]. In this regime, a thin liquid film is coated. The transition between the two regimes occurs at coating speeds on the order of 1 mm/s where the film thickness assumes a minimum. Directional crystallization is typically observed in this transition regime [1].

**Figure 2:**
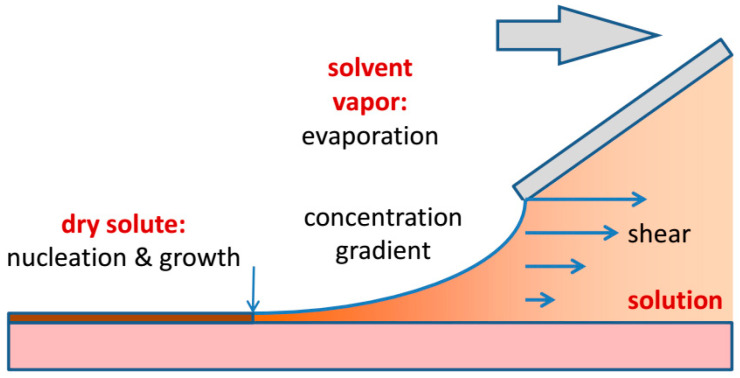
Processes during knife coating. At the edge of the coating blade, a well-defined shear field forms in the solution. As soon as the solution leaves the reservoir under the coating blade evaporation sets in. Eventually the supersaturation of the solution is high enough that solidification sets in at the drying line (arrow). Convective Marangoni currents may transport additional molecules to the drying line.

Looking at the meniscus region in more detail, it becomes clear that coating is a complex multi-scale non-equilibrium process, as shown in Figure 2. As the solution leaves the slot-die or the coating blade there is a well-defined shear field with a shear rate given by γ = *v*/*h*. In the meniscus region, the velocity gradient and, hence, the shear gradient diminishes, until the whole liquid column moves with the substrate speed. Evaporation of the solvent creates a concentration gradient perpendicular to the meniscus. Solidification at the drying line depletes the concentration of the solute in the solution and thus creates a concentration gradient at the drying line. This gradient will induce diffusive and convective transport of the solute to the drying line. When the solution reaches the critical supersaturation, nucleation will occur, either at the film–substrate interface, at the film surface, or both. Finally, the solution concentration, the volatility of the solvent, and the temperature of the substrate will determine how fast solvent evaporation and solute crystallization will occur.

Opinions on the role of shear during coating differ in the literature [3,4,10,11,14,15,16,17]. In order to shed light onto the importance of shear for directional crystallization, we reviewed a number of recent publications in which directional crystallization was achieved using a variety of coating methods and different amounts of shear. Chang et al. reported directional crystallization of TIPS-pn in slot-die coating using coating speeds between 0.2 and 1 mm/s and coating gaps between 10 µm and 90 µm [18]. Yildiz et al. studied zone casting of C8-BTBT and reported directional growth for a coating speed of 0.44 mm/s and concentrations ranging from 0.25 to 1 mg/mL in tetrahydrofuran [11]. Giri et al. used solution shearing of TIPS-pn at speeds of 0.4 to 1.6 mm/s [16]. In our own work based on knife coating using TIPS-pn [1], diF-TES-ADT [5], and C8-BTBT, we observed directional crystallization between 0.4 and 1 mm/s using a coating gap of 100 µm. Both high- and low-volatility solvents were used, and the substrate temperature could be adjusted between ambient temperature and 150 °C, which was chosen in order to optimize the film deposition for various solvents and solution concentrations. Headrick and coworkers achieved directional crystallization of TIPS-pn using a hollow pen writer at a speed of 0.1 mm/s and a relatively large coating gap of 0.6 mm [2]. Directional growth was also reported during dip coating by Park et al. [19]. Here, the coating speed was 0.7 mm/s and no mechanical barrier limited the initial film thickness. A similar result was obtained by Zhang et al. for C8-BTBT at speeds of 0.02–0.04 mm/s; they also studied different angles to withdraw the substrate from the solution [20]. Interestingly Lee et al. as well as Rivnay et al. obtained directional crystallization even in simple drop casting on a substrate inclined by 2° [21,22] when working with TIPS-pn and dicyanoperylene, respectively. In this case, surface tension prevented the solution drop from running off the substrate, while gravity gave rise to a wedge-shaped liquid layer. Without any mechanical action, the drying line moved slowly across the substrate as the solvent evaporated.

Hence, it appears that directional crystallization is an effect of the *speed of the drying line*, but independent of shear rate, since we found that the results were essentially independent of the coating gap. Indeed, rotational diffusion in small molecules is too fast to maintain any degree of orientation induced by the shear field at the coating blade by the time the drying line is reached. In contrast, conjugated polymers may retain some shear memory, particularly those with higher molecular weights.

## 3. A Hartman–Perdok Picture of Coating

In order to rationalize why directional crystallization seems to be a matter of the speed at which the drying line sweeps across the substrate, we will take a look at classic Hartman–Perdok theory [23,24]. Hartman and Perdok were interested in which crystallographic planes are expressed for macroscopic single crystals, i.e., the crystal habits. They concluded that stable facets are produced by the planes of slowest growth. These are typically the low-index Miller planes which feature smooth surfaces and a low density of docking sites. In contrast, vicinal surfaces have steps and kinks and display a much higher density of docking sites and thus grow faster; hence, such planes are not found in the habit of the crystal.

In the case of a moving drying line, we have the reverse situation: only planes with the *fastest* growing speed can follow the drying line, and such crystallites will grow in a specific direction. Other growth planes may form temporarily, but cannot keep up with the moving drying line and are cut off from further material transport. Thus, the drying line speed serves as a filter, allowing only the fastest growing planes to grow further. At lower coating speeds, multiple crystallographic planes can grow simultaneously, and eventually a 2D powder is formed with a random grain orientation. In the Landau–Levich regime at high coating speeds, nucleation occurs at random sites in the liquid film, often in the form of fast-growing spherulites, and again, a 2D powder is formed. Hence, there is only a small window of coating speeds where directional crystallization can occur.

**Figure 3:**
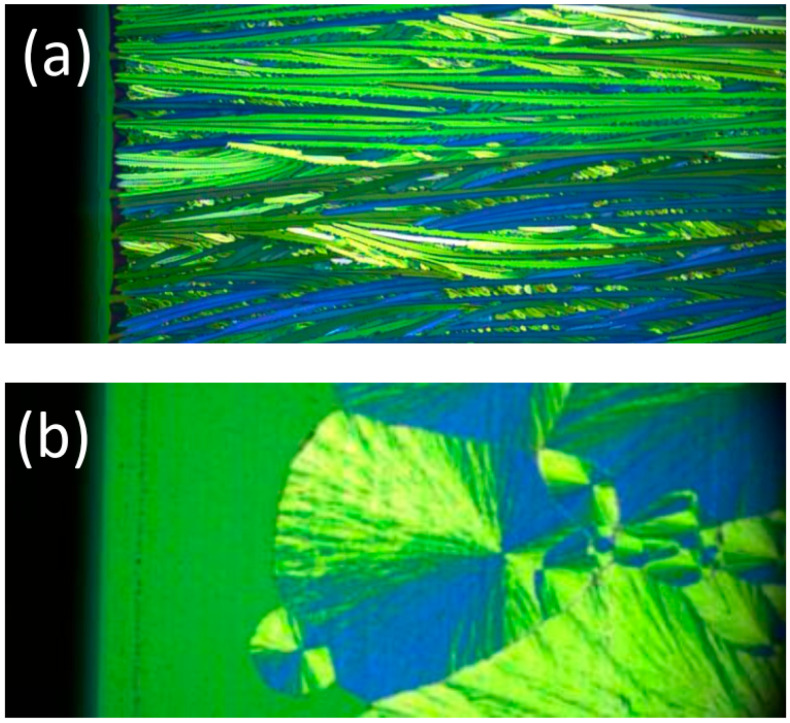
In situ cross-polarized micrographs of the coating regimes for TIPS-pn. (**a**) Directional growth in the transition regime at 0.4 mm/s. The crystallization of long needles starts right at the drying line. (**b**) Spherulitic growth in the Landau–Levich regime at 1.2 mm/s. A thin liquid film is deposited that eventually crystallizes. The coating blade was moving from right to left. The dark region on the left of both images is the shadow of the coating blade.

**Figure 4:**
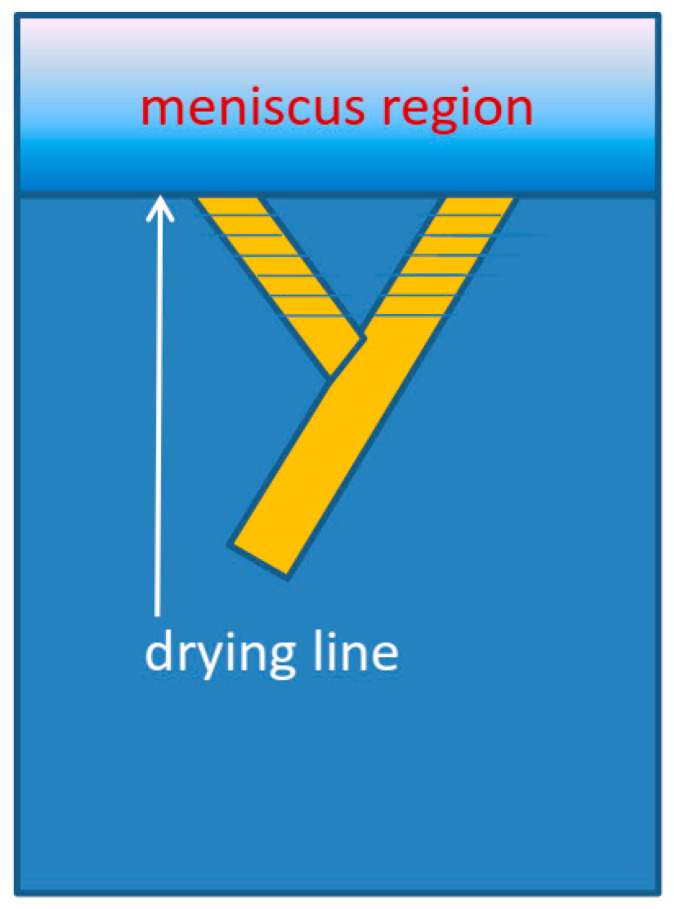
Proposed twinning mechanism. The fast-growing crystal planes of the crystallites emanating from the drying line and their slow-growth edges do not have to be perpendicular, especially for low-symmetry crystal lattices that are typical for conjugated molecules. Twinning limits the domain length as crystallites grow into each other.

If nucleation is high enough, multiple needle-like crystallites can form at the drying line. The sides of the needles will usually feature relatively stable, slow-growing, low-index planes. In low-symmetry triclinic or monoclinic lattices which are typically found for conjugated molecules, the growth front and the sides of the needles do not have to be perpendicular. Such a situation can give rise to twinning and the growth results in two needle orientations, as shown in Figure 3a and illustrated by Figure 4.

**Figure 5:**
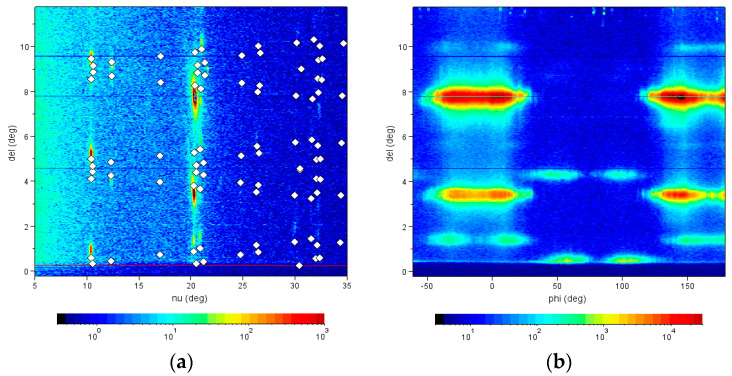
Survey scan and azimuth scan of a directionally grown diF-TES-ADT film. (**a**) The survey scan records the intensity as a function of the in-plane scattering angle *nu* and exit angle *del* while the sample is rotated azimuthally by 180°. This way, all reasonably strong film reflections can be found [26]. The dots indicate the expected positions of Bragg reflections [27] based on the published structure of diF-TES-ADT [28]. (**b**) For the azimuth scan around the surface normal, a fixed scattering angle *nu* of 20.3° was chosen, and features the strongest film reflections. The image shows pairs of reflections split by ±5°, indicative of directional growth with twinning.

Such a behavior was beautifully confirmed in high-resolution grazing-incidence X-ray diffraction images of a diF-TES-ADT film taken at the G2 station (see Figure 5) using a six-circle diffractometer with a high-resolution Soller slit and a linear diode array detector which simultaneously records the scattering intensity over a range of exit angles *del* from the sample surface [25]. A survey scan, in which the integrated intensity for each scattering angle *nu* of the sample was recorded while rotating the sample around the surface normal by 180°, revealed some strong diffraction intensities at the scattering angle *nu* of 20.3°. This scattering angle was further examined with an azimuthal scan that revealed split reflections by ±5°.

## 4. Synchronizing Clocks

In our experiments, a variety of conjugated molecules and solvents at different concentrations were used as well as various substrate temperatures and coating speeds. The substrate temperature controls the rate of evaporation as well as the diffusion rate to the drying line and the crystallization kinetics. The coating speed needs to be chosen properly so that the molecules can still attach to a growing crystal at the edge of the meniscus. The other factors are the initial concentration of the solute which determines when oversaturation is reached at a given substrate temperature as well as the material properties of the solute, solvent, and their combination. As such, the specific coating techniques is of less importance than the right combination of coating parameters.

Hence, achieving a high-quality directional crystallization seems to be a matter of synchronizing the different time scales involved, as defined by the coating speed, the crystallization speed, and the mass transport to the drying line. If the coating speed is too slow, multiple crystallites can grow and selectivity is lost [1]. If the coating speed is too fast, an intermediate liquid or amorphous film appears which subsequently nucleates spontaneously, forming spherulites [7,29]. Again, the directionality is lost. Fortuitously, the island of opportunity is not too sharply defined. A good guide is to look for the transition regime of film thickness versus coating speed. Thus, the experimenter has a good chance of locating the directional crystallization regime and then finetuning the coating parameters to achieve optimal performance.

## 5. Conclusions

Directional crystallization is an attractive way of optimizing device performance and repeatability in organic field-effect transistors [5,6,8,9,10,30,31]. We have shown here that directional crystallization can occur for a variety of coating techniques. We explain our findings by invoking classical crystallization theory and observing that under proper conditions, only the fastest growth planes can follow the drying line. Such growth can result in long single crystalline domains with dimensions up to centimeters. As such, the specific directional coating technique is less significant, provided that proper coating parameters are chosen. Due to the low symmetry lattices of most conjugated molecules, twinning is still possible which may limit the attainable length of single crystalline domains. The results reviewed here point out the importance of appropriate parameter control of the chosen coating process via in situ real-time characterization methods in order to optimize the structure and properties of crystalline thin films of small organic molecules.

## Data Availability

The data presented in this study are available from the authors upon reasonable request.
